# Detection and Validation of Circulating Endothelial Cells, a Blood-based Diagnostic Marker of Acute Myocardial Infarction

**DOI:** 10.1371/journal.pone.0058478

**Published:** 2013-03-04

**Authors:** Chuanyin Li, Qing Wu, Bo Liu, Yuting Yao, Ying Chen, Huili Zhang, Changqiang Wang, Jiumei Cao, Shengfang Ge

**Affiliations:** 1 Department of Ophthalmology, Ninth People's Hospital, Shanghai Jiao Tong University School of Medicine, Shanghai, China; 2 Department of Cardiology, Shanghai Ninth People's Hospital, Shanghai JiaoTong University School of Medicine, Shanghai, China; 3 Department of Geratology, Ruijin Hospital, Shanghai Jiaotong University School of Medicine, Shanghai, China; 4 Department of Biochemistry and Molecular Biology, Shanghai Jiaotong University School of Medicine, Shanghai, China; University of Padova Medical School, Italy

## Abstract

**Background:**

Circulating endothelial cells (CECs) are markers of vascular damage that have clinical relevance in many diseases, including acute myocardial infarction (AMI), and may be predictors of treatment responses. Herein, we investigated the diagnostic and prognostic value of CEC monitoring in AMI patients and a murine model.

**Methodology/Principal Findings:**

CECs were defined as Hoechst 33342^+^/CD45^−/^CD31^+^/CD146^+^/CD133^−^ in human blood samples and Hoechst 33342^+^/CD45^−/^CD31^+^/KDR^+^/CD117^−^ in murine samples. To evaluate the validity and variability of our CEC detection system, peripheral blood samples of vascular endothelial growth factor-treated athymic nude mice and AMI patients were collected and subjected to intra-assay analysis. CEC detection by flow cytometry and real-time PCR were compared. Blood samples were obtained from 61 AMI patients, 45 healthy volunteers and 19 samples of the original AMI patients accepted one month treatment, via flow cytometry and expressed as a percentage of peripheral blood mononuclear cells.

**Results:**

Our CEC detection method was validated and had limited variability. CEC concentrations were higher in AMI patients compared to healthy controls. One month post-treatment, CECs levels decreased significantly.

**Conclusions/Significance:**

CEC levels may be useful as a diagnostic and prognostic biomarker in AMI patients.

## Introduction

Circulating endothelial cells (CECs) are noninvasive markers of vascular damage, remodeling, and dysfunction [1], and considerable efforts have been directed at targeting the vascular components of malignant diseases. Currently, several methods are commonly used to identify CECs, including immunomagnetic isolation [2] and polychromatic flow cytometry [3]. CECs are present at very low levels in healthy subjects, whereas elevated levels have been reported in response to various pathological conditions, including acute myocardial infarction (AMI), coronary heart disease, infectious diseases, immunologic disorders, and cancers. [4], [5], [6] CECs can be used as a biomarker of these diseases, as they can potentially predict early responses to a course of treatment likely to benefit patients [7], [8]. Further, multiple studies have reported that CEC concentration is a potential surrogate marker of anti-angiogenic drug activity [2], [9], [10].

Many CEC antigens have been monitored by flow cytometry using monoclonal antibodies, but there are no antibodies specific to antigens that can discriminate CECs from peripheral blood cells. For examples, CD146 is an endothelial-specific marker [11], but it is also expressed by some mesenchymal cells and a subpopulation of activated lymphocytes [12]. Hence, only a multiparametric, concurrent investigation using several antibodies can discriminate CECs from peripheral blood cells. The first step in such analyses is to exclude hematopoietic cells using the pan-hematopoietic marker CD45, and then confirm the endothelial nature of the remaining CD45-negative cells using two or more endothelial markers, such as CD146, CD31, or kinase insert domain receptor (KDR, also known as vascular endothelial growth factor receptor 2, VEGFR2).

Previous studies have established that CECs may be elevated in murine angiogenesis models, which can decrease following administration of antiangiogenic agents [13]. In murine models, the preclinical number of CEC correlates with angiogenic effects. Therefore, we hypothesize that CECs originating from endothelium sloughed into the circulation may increase after antiangiogenic administration. Because CECs occur during pathological conditions including vacuities, infection, and myocardial infarction [4], [14], [15], [16], a method is needed to more accurately identify CEC populations to reflect the angiogenic microenvironment. CEC levels have also been investigated using a previously established model of VEGF-induced mice [17]. Hence, CECs present a useful marker for detecting angiogenesis or monitoring therapy responses that affect vasculature [18]. Myocardial infarction (MI) is a leading cause of death worldwide, and CEC levels are significantly elevated in AMI [4]; however, changes in CEC concentrations post-treatment have not been reported. Therefore, in the present study, we evaluated the use of CECs as a blood-based biomarker of cardiovascular diseases via flow cytometry and validated this method using known antigenic markers.

## Materials and Methods

### Endothelial Cell Line Culture

MS-1 cells (The American Type Culture Collection, Manassas, VA, USA), a transformed murine endothelial cell line isolated from pancreatic islets of C57BL/6 mice, were used as a positive control and maintained in Dulbecco’s modified Eagle’s medium (Invitrogen, Carlsbad, CA, USA) supplemented with 10% fetal bovine serum (HyClone, Logan, UT, USA), 50 µg/ml of gentamicin, 5 µg/ml of transferrin, and 10 ng/ml of epidermal growth factor.

### Animal Models

Ten athymic nude mice (8–10-week-old) were purchased from the Shanghai Laboratory Animal Center (Shanghai, China) and housed under pathogen-free conditions. All animal experiments were performed in accordance with institutional guidelines for animal care at Shanghai Jiao Tong University and approved by Animal Care and Use Committee of Shanghai Jiao Tong University. Every nude mouse was injected daily with 10 µg of recombinant human VEGF (National Cancer Institute, Biological Resources Branch, Frederick, MD, USA) for 5 days. Blood was collected by retro-orbital puncture and anticoagulated using ethylenediaminetetraacetic acid (EDTA).

### Flow Cytometry

**Figure 1:**
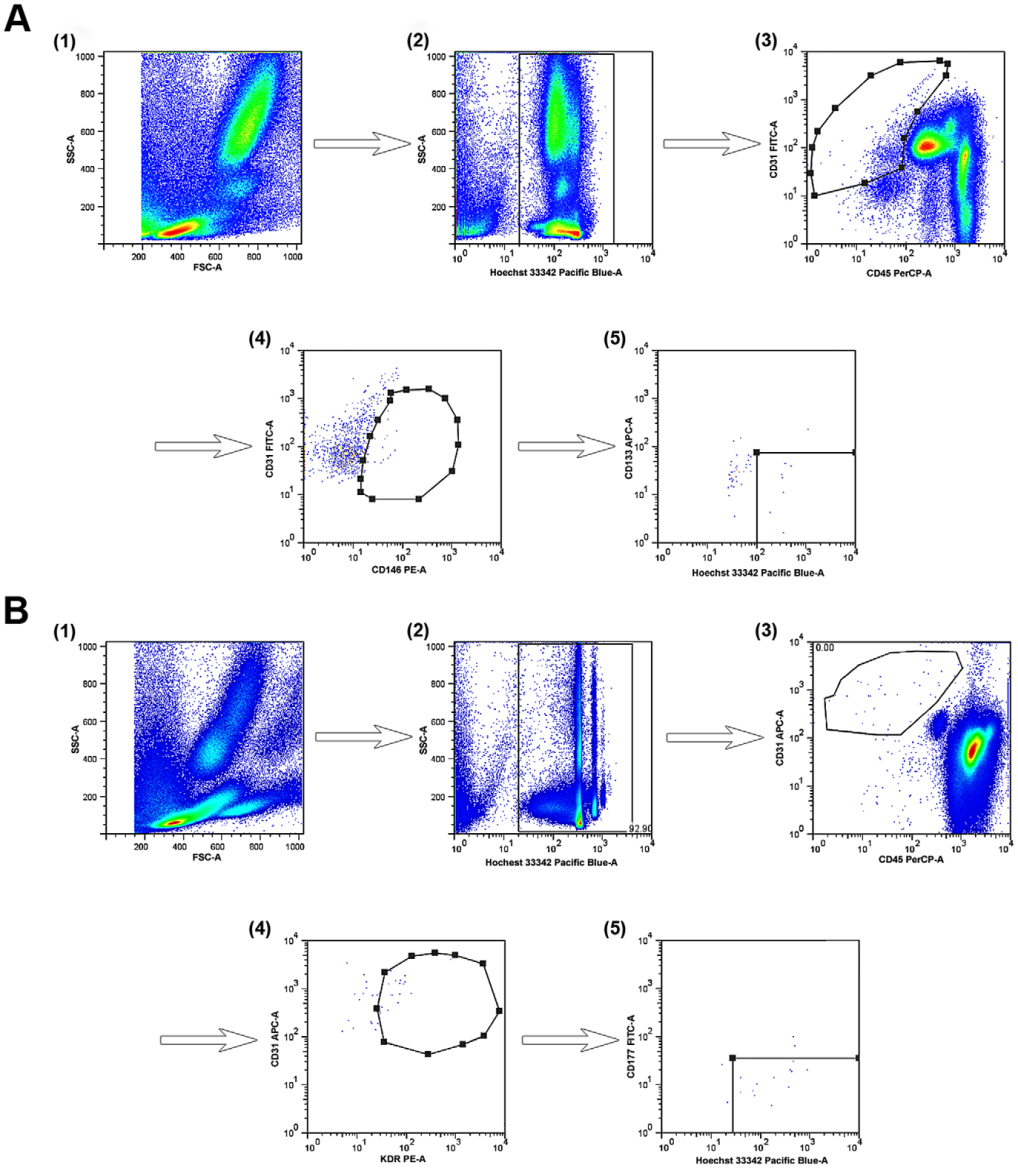
Gating strategy to characterize circulating endothelial cells (CECs) by surface markers. (**A**) Human CEC Panel: (**1**) whole fresh blood; (**2**) select Hoechst33342+ cells; (**3**) CD45−/CD31+ cells; (**4**) CD31+/CD146+ co-expression; and (**5**) select CD133−/Hoechst 33342+ cells (CECs). (**B**) Mouse CEC Panel: (**1**) whole fresh blood; (**2**) select Hoechst 33342+ cells; (**3**) select CD45−/CD31+ cells; (**4**) CD31+/KDR+ co expression; and (**5**) select CD117−/Hoechst 33342+ cells (CECs).

Whole human blood was drawn from healthy donors and AMI patients in 2 ml EDTA-coated tubes. For the mouse study, after anesthesia, fresh blood was collected via heart puncture into 2-ml EDTA-coated tubes, mixed well, and stained. Red blood cells were lysed using BD fluorescence-activated cell sorting (FACS)™ Lysing Solution (BD Biosciences, San Jose, CA, USA), per the manufacturer's instructions. The following conjugated antibodies (BD Biosciences) were used for detection of CECs in mouse peripheral blood: anti-mouse CD45-PerCP (catalog no.: 553083), KDR-PE (catalog no.: 555308), CD31-APC (platelet/endothelial cell adhesion molecule-1; catalog no.: 551262), and CD117-FITC (catalog no.: 553354). The following conjugated antibodies were used for detection of human CECs, CD31-FITC (catalog no.: 11-0319-73; eBiosciences, San Diego, CA, USA), CD45-PerCP (catalog no.: 555484, BD Biosciences), CD146-PE (catalog no.: 550315, BD Biosciences), and CD133-APC (catalog no.: 17-1338-42, eBiosciences). To identify nuclear cells, Hoechst 33342 stain (Invitrogen) was used as previously described. Data acquisition was performed on a FACS-Canto II flow cytometer (BD Biosciences) after adjusting for antibodies bound to BD-compensation particles. For the CEC panel, the lymphocyte population was gated in the forward scatter-side scatter (FSC-SSC) detector and 0.8–1.2×106 mononuclear cells were acquired. The subpopulations were identified according to their specific surface staining: human CECs: Hoechst 33342^+^/CD45^−/^CD31^+^/CD146^+^/CD133^−^ and mouse CECs: Hoechst 33342^+^/CD45^−/^CD31^+^/KDR^+^/CD117^−^. The flow cytometry protocols are shown in **Figures 1A and 1B**. Data evaluation was carried out using FACS-Diva software (BD Biosciences), and data analysis was performed using FlowJo version 7.2.2 software (www.flowjo.com/download/index.html). Statistical data were imported from the FlowJo program and analyzed with Statistical Package for the Social Sciences (SPSS) version 17.0 (SPSS, Inc., Chicago, IL, USA) to determine the standard deviation and the coefficient of variation (CV) using the formula CV = (standard deviation/mean×100%).

### Intra-assay Variability Analysis

To assess the technical variability of flow cytometry analysis, an intra-assay was performed on 5 VEGF-treated mice and 5 AMI patients. Briefly, peripheral blood samples were divided into 8 replicates and analyzed by flow cytometry. Intra-assay variability was calculated as the CV values of 8 replicates.

### Measurement of CECs by Real-time PCR

Blood samples (2 ml) from 5 healthy mice and 5 VEGF-treated mice were obtained and 1 ml was subjected to flow cytometry analysis, and the other 1 ml from the same tube was mixed with 15 ml of erythrocyte lysis buffer (0.899% (w/v) ammonium chloride, 0.1% (w/v) potassium bicarbonate, and 0.0037% (w/v) EDTA, pH 7.3) and incubated for 10 min at room temperature (RT). After the samples were centrifuged for 5 min at 500 g, the lysis buffer was removed and the cell pellet was resuspended in 350 µl of RLT buffer (Qiagen, Hilden, Germany). RNA was isolated using the RNeasy Kit (Qiagen, USA) according to the manufacturer’s instructions. Total RNA (2,500 ng) was reverse-transcribed to complementary DNA (cDNA) using the PrimeScript 1st Strand cDNA Synthesis kit (TaKaRa Bio, Inc., Shinga, Japan) following the manufacturer’s instructions. For the amplification of KDR (KDR sense 5′-GAACCTGACTATCCGCAGGG-3′, and antisense 5′-AGGAGCCAGAAGAACATGGC-3′; GRAPDH sense 5′-TGATGGG TGTGAACCACGAG-3′, and antisense 5′- ATCACGCCACAGCTTTCCAG-3′), 1 µl of cDNA was added to SYBR Green PCR Master Mix (Qiagen, USA). PCR was performed on ABI 7500 Real-Time PCR System (Applied Biosystems, Foster City. CA, USA) using the following thermal settings: one cycle of 10 min at 95°C, and 40 cycles of 15 s at 95°C, and 1 min at 60°C. Relative mRNA expression was calculated with the 2^−ΔΔCt^ method [19], and the results were compared to the flow cytometry CEC analysis.

### Daily Variability in CEC Percentage

To assess the daily variability in the relative CEC concentration of one AMI patient, peripheral blood was taken at different time points (0, 24, 48, and 72 h) and each sample was analyzed in 4 replicates. For the mouse study, all blood samples were collected at the same time and stored at RT due to bleeding affect the CEC concentrations. The samples were assayed at different time points (0, 24, 48, and 72 h) and each analyzed in 4 replicates.

### Patients

The research was carried out according to the principles of the Declaration of Helsinki. Informed consents written by participants were obtained and this study was approved by Ethics Committee of the Shanghai Ninth People's Hospital, Shanghai JiaoTong University School of Medicine. The patient data, which are contained within this article, were obtained by a hospital-based doctor at Shanghai Ninth People's Hospital and Ruijin Hospital, Shanghai JiaoTong University School of Medicine. Permission to use these data in this report has been obtained from all the subjects who participated in this study. This prospective study included consecutive AMI patients treated at the Ninth People’s Hospital and Ruijin Hospital, Shanghai Jiaotong University School of Medicine (Shanghai, China). AMI was diagnosed via angiocardiography, biochemical markers, and cardiac enzyme tests.

### CEC Analysis

The peripheral blood samples were collected from patients who met the AMI criteria. The percentage of CECs was determined by flow cytometry. One month post-treatment, blood samples were collected again from some of these original AMI patients.

### Statistical Analysis

The results are presented as means ± standard deviation (SD). The difference between two groups was analyzed using the two-tailed Student’s *t*-test and a confidence level of *p*<0.05 was considered statistically significant, *p*<0.01 was considered statistically very significant.

## Results

### Intra-assay Variability Analysis

**Figure 2:**
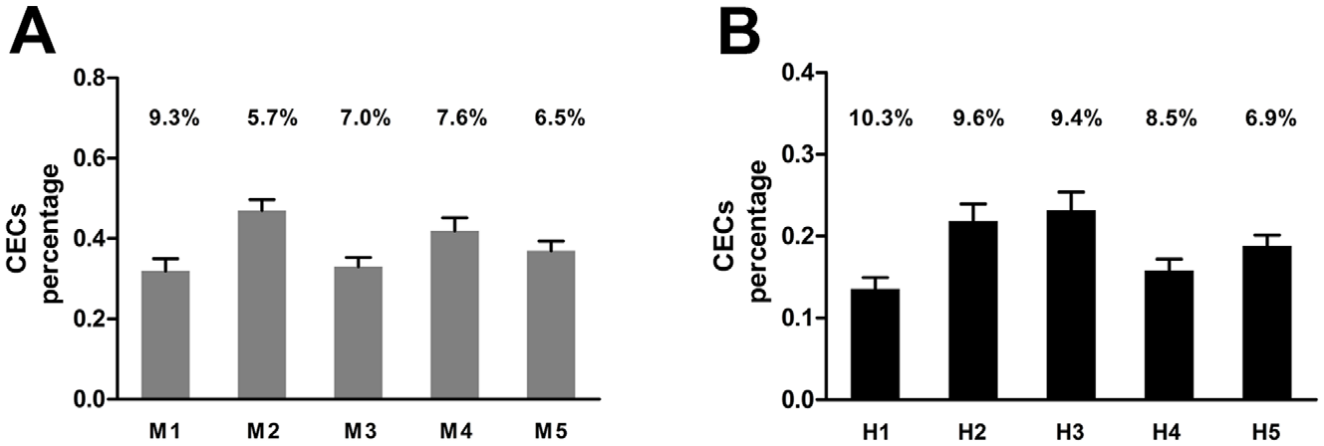
Intra-assay variability analysis of CECs in murine and human peripheral blood. CEC counts are expressed as a percentage of peripheral blood mononuclear cells. Intra-assay variability is presented as the CV value of 8 replicates, calculated as (standard deviation/mean)×100%. (**A**) In 5 VEGF-treated mice (M1–5), the CV values were 9.3% (M1), 5.7% (M2), 7.0% (M3), 7.6% (M4), and 6.5% (M5). (**B**) In 5 AMI patients (H1–5), the CV values were 10.3% (H1), 9.6% (H2), 9.4% (H3), 8.5% (H4), and 6.9% (H5).

An intra-assay was performed using samples from 5 VEGF-treated mice (M1–5) and 5 AMI patients (H1–5). As shown in **Figure 2**, CEC counts are expressed as the percentage of peripheral blood mononuclear cells and the CV value was calculated as (standard deviation/mean)×100%. The CV values of the mice samples were 9.3% (M1), 5.7% (M2), 7.0% (M3), 7.6% (M4), and 6.5% (M5), whereas the CV values of the AMI patients were 10.3% (H1), 9.6% (H2), 9.4% (H3), 8.5% (H4), and 6.9% (H5). In all samples, the CV values were <11%, suggesting that the variability of flow cytometry analysis was limited and our CECs detection system was stable.

### Measurement Consistency of CECs Detected by Flow Cytometry and Real-time PCR

**Figure 3:**
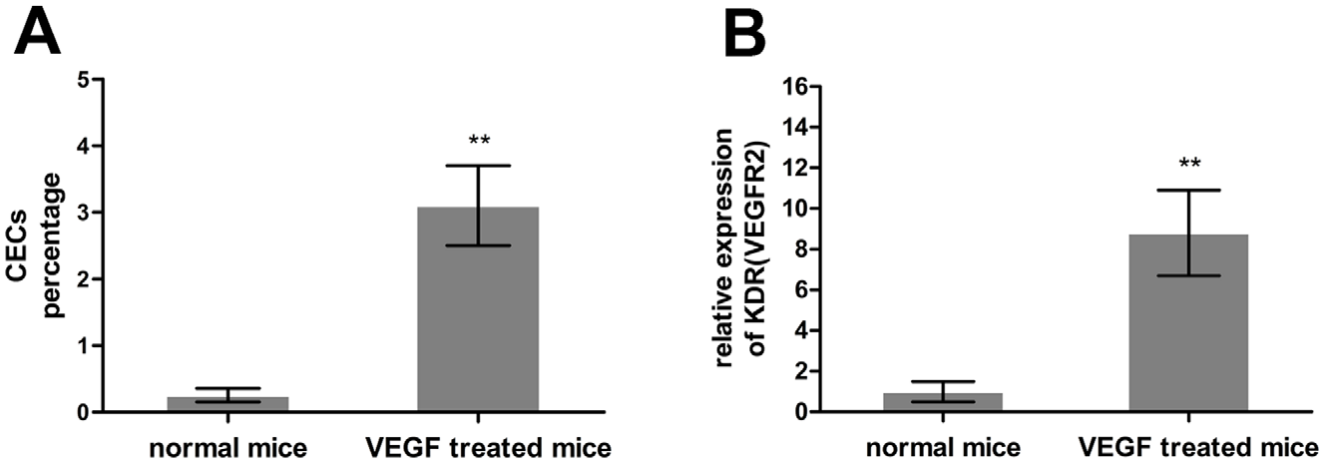
CECs in mouse peripheral blood detected by flow cytometry and real-time PCR. (**A**) CEC percentage detected by flow cytometry. (**B**) CEC concentration detected by real-time PCR. KDR is an endothelial cell marker. (*n* = 5/group, **p<0.01).

The mean percentage of CECs in blood samples from normal and VEGF-treated mice was 0.256 and 3.104%, respectively (**Figure 3A**). Relative KDR mRNA expression was measured in blood samples from VEGF-treated mice and healthy controls to characterize CEC concentrations. As shown in **Figure 3B**, VEGF treatment induced an 8.8-fold increase in KDR expression. The increased CEC percentage and relative KDR expression in VEGF-treated mice were in agreement.

### Daily Variability in CEC Concentration

**Figure 4:**
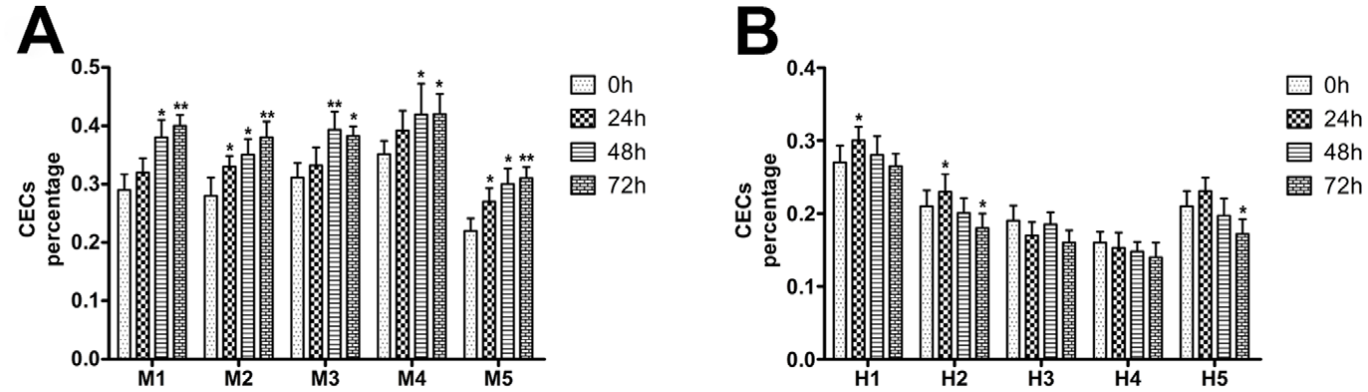
Daily variability in CEC concentration. (**A**) Peripheral blood samples of VEGF-treated mice were collected at the same time and the CEC concentration was determined at different time points (0, 24, 48, and 72 h). (**B**) Peripheral blood samples of AMI patients were collected at different time points (0, 24, 48, and 72 h) and the CEC concentrations were determined. The error bar represents the standard deviation of 4 replicates. (*p<0.05, **p<0.01).

For the human study, peripheral blood samples were collected at different time points (0, 24, 48, and 72 h). As shown in **Figure 4B**, the percentage of CECs reached a maximum value at 24 h. For the mouse study, the blood samples were collected at the same time and stored at RT. The percentage of CECs was measured at different time points (0, 24, 48, and 72 h). As shown in **Figure 4A**, the percentage of CECs elevated quickly with time.

### Elevated CEC Levels in AMI Patients

**Figure 5:**
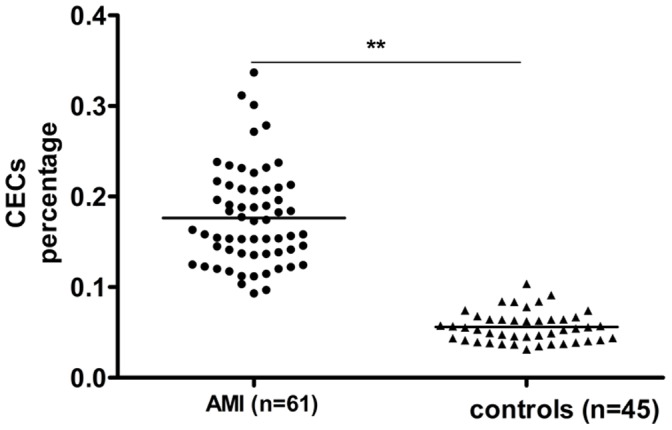
CEC concentrations in AMI patients and healthy controls. CEC counts are expressed as the percentage of peripheral blood mononuclear cells. **p<0.01.

A total of 106 subjects were recruited for the present study between December 2011 and October 2012, which included 61 AMI patients and 45 age-matched healthy subjects. **Table 1** summarizes the characteristics of the study groups. As shown in **Figure 5**, the percentage of CECs in AMI patients was significantly higher than that in healthy subjects (**Figure 5**), which was consistent with the results of biochemical marker analysis and cardiac enzyme tests (**Table 2**).

**Table 1:** Characteristics of patients.

Parameters	AMI (n = 61)	controls (n = 45)
Age(years)	58.5±12.3	56.4±17.2
Male/female(n/n)	44/17	31/14
Diabetes mellitus, n (%)	28(46%)	16(35%)
Hypertension, n (%)	34(56%)	14(31%)
Current smoking, n (%)	45(73%)	23(51%)

AMI, acute myocardial infarction.

**Table 2:** Biochemical markers and cardiac enzymes.

Markers	AMI(n = 61)	Post-treatment(n = 19)	Controls (n = 45)	P1	P2	P3
WBC (*10^9^/L)	7.9±3.1	6.5±2.1	6.3±2.4	*	*	−
Mb (ng/ml)	197.5±57.9	52±19.4	48.4±28.1	*	*	−
cTnl (ng/ml)	0.64±1.57	0.03±0.17	0.02±0.05	**	**	−
CK (IU/L)	347.5±689.3	154.5±47.2	139.2±37.2	*	*	−
CK-MB (IU/L)	10.4±27.9	4.2±2.7	3.8±1.9	*	*	−
AST (IU/L)	89.1±123.7	17.2±12.4	15.8±13.1	**	**	−
LDH (IU/L)	397.9±642.5	147.4±64.8	161±56.8	**	**	−

AMI, acute myocardial infarction; WBC, white blood cell; Mb, Myoglobin; cTnl, troponin; CK, creatine kinase; CK-MB, the MB isoenzyme of creatine kinase; AST, aspartate aminotransferase; LDH, lactate dehydrogenase.

* p<0.05,

** p<0.01,

“−” no statistically significant difference.

### Changes in CEC Percentage Post-treatment

**Figure 6:**
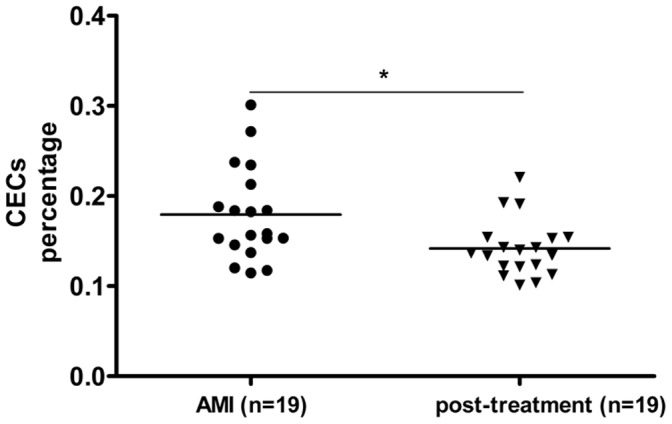
Changes in CEC concentrations post-treatment. CEC concentrations are expressed as the percentage of peripheral blood mononuclear cells. *p<0.05.

One month post-treatment, the peripheral blood of 19 AMI patients was collected again, and as the other 42 patients have left our hospital and we could not obtain further blood samples. As shown in **Figure 6**, the CEC percentage post-treatment decreased significantly compared with pre-treatment, whereas biochemical markers and cardiac enzymes were at normal levels.

## Discussion

CEC concentration is a biomarker of endothelial damage, which has been correlated to other markers of endothelial function, including flow-mediated dilation, von Willebrand factor, and tissue plasminogen activator levels [20], [21], [22]. CEC markers are diverse [22], [23], human CECs are defined as Hoechst 33342+/CD45−/CD31+/CD146+/CD133−, whereas mouse CECs are Hoechst 33342+/CD45−/CD31+/KDR+/CD117− in our study. The intra-assay analysis indicated that our detection system had limited variability.

CEC concentrations were significantly (8.8-fold) increased in VEGF-treated mice compared to normal controls. KDR (VEGFR2) is a marker of CEC [23], detected by real-time PCR was consistent with CEC percentage detected by flow cytometry, suggesting that our detection system was valid.

Cardiovascular disease is a leading cause of morbidity and mortality worldwide and methods for assessing endothelial function are well established. The measurement of immunologically defined CECs in peripheral blood is gaining ground as an important technique for assessment of endothelial injury [1], [4]. In this study, we found that CEC levels were elevated in peripheral blood samples from AMI patients; hence, CECs can act as a blood-based biomarker of cardiovascular diseases and pose a good target for MI pharmaceutical development.

CECs may also have clinical relevance in many other diseases, as CEC concentration was increased in cancer patients and correlated with tumor progression [24], [25], [26]. Further, CEC quantification can identify patients who might benefit from angiogenesis inhibitors and be used to monitor treatment response [27], [28]. In the current study, CEC concentrations decreased significantly 1 month post-treatment compared with pre-treatment, but still remained higher than in healthy controls. However, biochemical markers and cardiac enzymes were at normal levels, indicating that CECs present a useful prognostic marker.

In summary, the CEC concentration in peripheral whole blood is quite low; therefore, the interpretation of results should be made cautiously. Since the performance of the standardized flow cytometry method for CEC quantification was stable, it is suitable for use in clinical applications. In murine models, CEC levels increased following VEGF-treatment compared to normal control mice. Therefore, CEC levels provide information specific to angiogenesis and present a blood-based biomarker detection system to clinically monitor drug efficacy. However, the role of endothelial markers in cardiovascular disease remains to be addressed in future mechanistic and prospective clinical studies using other tissues. Also, new markers and methods must be developed to more accurately measure the angiogenic process. Furthermore, anti-angiogenesis therapy can limit angiogenesis, which can be monitored via CEC concentrations using the immunomagnetic separation method [2]. In future studies, CEC monitoring can be used to monitor targets of anti-angiogenesis therapies.

## References

[1] Smadja DM, Gaussem P, Mauge L, Israel-Biet D, Dignat-George F, et al (2009) Circulating endothelial cells: a new candidate biomarker of irreversible pulmonary hypertension secondary to congenital heart disease. Circulation 119: 374–381. 19139384 10.1161/CIRCULATIONAHA.108.808246

[2] Woywodt A, Blann AD, Kirsch T, Erdbruegger U, Banzet N, et al (2006) Isolation and enumeration of circulating endothelial cells by immunomagnetic isolation: proposal of a definition and a consensus protocol. J Thromb Haemost 4: 671–677. 16460450 10.1111/j.1538-7836.2006.01794.x

[3] DuBois SG, Stempak D, Wu B, Mokhtari RB, Nayar R, et al (2012) Circulating endothelial cells and circulating endothelial precursor cells in patients with osteosarcoma. Pediatr Blood Cancer 58: 181–184. 21319292 10.1002/pbc.23046 PMC3070958

[4] Damani S, Bacconi A, Libiger O, Chourasia AH, Serry R, et al (2012) Characterization of circulating endothelial cells in acute myocardial infarction. Sci Transl Med 4: 126ra133. 10.1126/scitranslmed.3003451 PMC3589570 22440735

[5] Kondo S, Ueno H, Hashimoto J, Morizane C, Koizumi F, et al (2012) Circulating endothelial cells and other angiogenesis factors in pancreatic carcinoma patients receiving gemcitabine chemotherapy. BMC Cancer 12: 268. 22731825 10.1186/1471-2407-12-268 PMC3437212

[6] Ring A, Smith IE, Dowsett M (2004) Circulating tumour cells in breast cancer. Lancet Oncol 5: 79–88. 14761811 10.1016/S1470-2045(04)01381-6

[7] Asahara T, Murohara T, Sullivan A, Silver M, van der Zee R, et al (1997) Isolation of putative progenitor endothelial cells for angiogenesis. Science 275: 964–967. 9020076 10.1126/science.275.5302.964

[8] Shaked Y, Kerbel RS (2007) Antiangiogenic strategies on defense: on the possibility of blocking rebounds by the tumor vasculature after chemotherapy. Cancer Res 67: 7055–7058. 17671170 10.1158/0008-5472.CAN-07-0905

[9] Nanda A, Karim B, Peng Z, Liu G, Qiu W, et al (2006) Tumor endothelial marker 1 (Tem1) functions in the growth and progression of abdominal tumors. Proc Natl Acad Sci U S A 103: 3351–3356. 16492758 10.1073/pnas.0511306103 PMC1413931

[10] Bertolini F, Paul S, Mancuso P, Monestiroli S, Gobbi A, et al (2003) Maximum tolerable dose and low-dose metronomic chemotherapy have opposite effects on the mobilization and viability of circulating endothelial progenitor cells. Cancer Res 63: 4342–4346. 12907602

[11] Malyszko J, Malyszko JS, Brzosko S, Wolczynski S, Mysliwiec M (2004) Adiponectin is related to CD146, a novel marker of endothelial cell activation/injury in chronic renal failure and peritoneally dialyzed patients. J Clin Endocrinol Metab 89: 4620–4627. 15356072 10.1210/jc.2004-0387

[12] Elshal MF, Khan SS, Takahashi Y, Solomon MA, McCoy JP Jr (2005) CD146 (Mel-CAM), an adhesion marker of endothelial cells, is a novel marker of lymphocyte subset activation in normal peripheral blood. Blood 106: 2923–2924. 16204154 10.1182/blood-2005-06-2307

[13] Mancuso P, Calleri A, Cassi C, Gobbi A, Capillo M, et al (2003) Circulating endothelial cells as a novel marker of angiogenesis. Adv Exp Med Biol 522: 83–97. 12674213 10.1007/978-1-4615-0169-5_9

[14] Shet AS, Aras O, Gupta K, Hass MJ, Rausch DJ, et al (2003) Sickle blood contains tissue factor-positive microparticles derived from endothelial cells and monocytes. Blood 102: 2678–2683. 12805058 10.1182/blood-2003-03-0693

[15] St Croix B, Rago C, Velculescu V, Traverso G, Romans KE, et al (2000) Genes expressed in human tumor endothelium. Science 289: 1197–1202. 10947988 10.1126/science.289.5482.1197

[16] Carmeliet P (2005) Angiogenesis in life, disease and medicine. Nature 438: 932–936. 16355210 10.1038/nature04478

[17] Beaudry P, Force J, Naumov GN, Wang A, Baker CH, et al (2005) Differential effects of vascular endothelial growth factor receptor-2 inhibitor ZD6474 on circulating endothelial progenitors and mature circulating endothelial cells: implications for use as a surrogate marker of antiangiogenic activity. Clin Cancer Res 11: 3514–3522. 15867254 10.1158/1078-0432.CCR-04-2271

[18] Bertolini F, Shaked Y, Mancuso P, Kerbel RS (2006) The multifaceted circulating endothelial cell in cancer: towards marker and target identification. Nat Rev Cancer 6: 835–845. 17036040 10.1038/nrc1971

[19] Yao Y, Wang L, Zhang H, Wang H, Zhao X, et al (2012) A Novel Anticancer Therapy That Simultaneously Targets Aberrant p53 and Notch Activities in Tumors. PLoS One 7: e46627. 23071601 10.1371/journal.pone.0046627 PMC3468572

[20] Doroszewski W, Wlodarczyk Z, Strozecki P, Manitius J, Grabarczyk E, et al (2007) [Assessment of plasminogen activator inhibitor-1 and von Willebrand factor as a markers of endothelial function in patients with end-stage kidney disease after allotransplantation during a one-year follow-up]. Pol Arch Med Wewn 117: 213–220. 18030870

[21] Rodriguez-Osorio X, Sobrino T, Brea D, Martinez F, Castillo J, et al (2012) Endothelial progenitor cells: a new key for endothelial dysfunction in migraine. Neurology 79: 474–479. 22815557 10.1212/WNL.0b013e31826170ce

[22] De Palma M, Venneri MA, Galli R, Sergi Sergi L, Politi LS, et al (2005) Tie2 identifies a hematopoietic lineage of proangiogenic monocytes required for tumor vessel formation and a mesenchymal population of pericyte progenitors. Cancer Cell 8: 211–226. 16169466 10.1016/j.ccr.2005.08.002

[23] Sun X, Cheng L, Duan H, Lin G, Lu G (2012) Characterization and comparison of embryonic stem cell-derived KDR+ cells with endothelial cells. Microvasc Res 84: 149–154. 22706170 10.1016/j.mvr.2012.06.003

[24] Mancuso P, Burlini A, Pruneri G, Goldhirsch A, Martinelli G, et al (2001) Resting and activated endothelial cells are increased in the peripheral blood of cancer patients. Blood 97: 3658–3661. 11369666 10.1182/blood.v97.11.3658

[25] Rowand JL, Martin G, Doyle GV, Miller MC, Pierce MS, et al (2007) Endothelial cells in peripheral blood of healthy subjects and patients with metastatic carcinomas. Cytometry A 71: 105–113. 17226859 10.1002/cyto.a.20364

[26] Beerepoot LV, Mehra N, Vermaat JS, Zonnenberg BA, Gebbink MF, et al (2004) Increased levels of viable circulating endothelial cells are an indicator of progressive disease in cancer patients. Ann Oncol 15: 139–145. 14679134 10.1093/annonc/mdh017

[27] Calleri A, Bono A, Bagnardi V, Quarna J, Mancuso P, et al (2009) Predictive Potential of Angiogenic Growth Factors and Circulating Endothelial Cells in Breast Cancer Patients Receiving Metronomic Chemotherapy Plus Bevacizumab. Clin Cancer Res 15: 7652–7657. 19996223 10.1158/1078-0432.CCR-09-1493

[28] Fleitas T, Martinez-Sales V, Vila V, Reganon E, Mesado D, et al (2012) Circulating endothelial cells and microparticles as prognostic markers in advanced non-small cell lung cancer. PLoS One 7: e47365. 23077602 10.1371/journal.pone.0047365 PMC3471832

